# Impact of post-resistance molecular retesting strategies on treatment timeliness and clinical outcomes in EGFR-mutant advanced NSCLC: a real-world study

**DOI:** 10.3389/fonc.2026.1969822

**Published:** 2026-09-25

**Authors:** Wenting Zhang, Feng Cheng, Ping Lin, Jinhe Xu, Ying Chen, Jiaqi Meng, Xiulian Huang, Wen Wen, Zhongquan Zhao, Yingfang Song, Lei Zhang, Zongyang Yu

**Affiliations:** 1 Fuzong Clinical Medical College of Fujian Medical University, Fuzhou, China; 2 Department of Pulmonary and Critical Care Medicine, Fuzong Clinical Medical College of Fujian Medical University & The 900th Hospital of Joint Logistic Support Force, People’s Liberation Army (PLA), Fuzhou, China

**Keywords:** circulating tumor DNA, EGFR mutation, non-small cell lung cancer, real-world study, time to treatment initiation

## Abstract

**Objective:**

To evaluate the association of ctDNA-based molecular retesting with treatment timeliness and clinical outcomes in patients with advanced EGFR-mutant NSCLC after first-line EGFR-TKI resistance.

**Patients and methods:**

We retrospectively analyzed patients with advanced EGFR-mutant NSCLC who had radiologically confirmed progression after first-line EGFR-TKIs between 2019 and 2024. Patients were classified as no retesting (n=86), tissue NGS (n=94), or ctDNA testing (n=49). Primary endpoints were second-line progression-free survival (2L-PFS) and overall survival from initiation of second-line therapy (2L-OS). Secondary endpoints included time to treatment initiation (TTI) and treatment patterns. Survival was analyzed using Kaplan–Meier and log-rank methods; Cox models were used for exploratory subgroup analyses. A 1:1:1 propensity score–matched analysis and sensitivity analyses restricted to patients who initiated second-line therapy within 28 or 30 days after progression were performed.

**Results:**

Among 229 patients, median TTI was shorter with ctDNA than tissue NGS (11.0 vs. 20.5 days, P < 0.001). Subsequent targeted therapy was most frequently used in the ctDNA group (95.9%), followed by the tissue NGS (89.4%) and no-retesting groups (81.4%; P = 0.039). After matching, median 2L-PFS was 5.0, 9.0, and 9.0 months in the no-retesting, tissue NGS, and ctDNA groups, respectively (overall P = 0.005). Median 2L-OS was 12.0, 20.0, and 19.0 months, respectively (overall P = 0.074). No significant differences in 2L-PFS or 2L-OS were observed between ctDNA and tissue NGS, and findings were consistent in 28- and 30-day sensitivity analyses.

**Conclusions:**

Compared with tissue NGS, ctDNA testing was associated with shorter TTI, with no significant differences in 2L-PFS or 2L-OS. The ctDNA group had the highest observed targeted therapy use.

## 1 Introduction

Acquired resistance remains a major limitation of first-line EGFR-TKI therapy in advanced EGFR-mutant NSCLC (1, 2). With the widespread use of third-generation EGFR-TKIs, resistance mechanisms have become increasingly complex and heterogeneous (3, 4), making molecular retesting essential for guiding subsequent therapy selection (5). In practice, however, tissue re-biopsy is not always feasible. Lesions may be difficult to access, procedures may carry risk, tissue yield may be inadequate, and the overall process may prolong the interval before subsequent therapy can begin (6, 7).

ctDNA testing provides a blood-based approach that can be repeated with less procedural burden than tissue biopsy (6–8). Plasma-based genotyping has also been reported to shorten molecular testing turnaround time compared with tissue-based testing (9). In ACCELERATE, median turnaround time was 7 days for plasma ctDNA testing and 23 days for tissue NGS, and early ctDNA testing was associated with earlier treatment initiation (9). In addition to its time advantage, ctDNA testing may complement tissue testing by identifying actionable alterations when tissue is insufficient or unavailable (6, 7). Because ctDNA may be released from multiple disease sites, plasma profiling may capture molecular alterations not represented in a single-site tissue biopsy (10, 11).

Despite these advantages, whether the shorter turnaround time of ctDNA testing translates into earlier treatment initiation and more efficient delivery of subsequent therapy in routine clinical practice remains insufficiently characterized (12). Most existing studies have focused on testing turnaround time, technical concordance, and actionable alteration detection (7, 9, 13), with limited real-world evidence linking post-resistance testing strategies to subsequent treatment delivery and clinical outcomes. To address this gap, we compared no retesting, tissue NGS, and plasma ctDNA testing with respect to time to treatment initiation, post-resistance molecular profiles, subsequent treatment patterns, 2L-PFS, and 2L-OS in patients with advanced EGFR-mutant NSCLC after progression on first-line EGFR-TKIs.

## 2 Materials and methods

### 2.1 Study population and patient selection

This study included patients with advanced EGFR-mutant NSCLC who were diagnosed and treated at a single tertiary hospital between January 2019 and December 2024. This study was approved by the Ethics Committee of the 900th Hospital of the Joint Logistic Support Force of China (No. 2025-054) and was conducted in accordance with the Declaration of Helsinki. The requirement for informed consent was waived by the ethics committee because of the retrospective study design. A total of 794 patients were initially screened, and 229 eligible patients were ultimately included. The patient selection process is illustrated in **Figure 1**.

**Figure 1:**
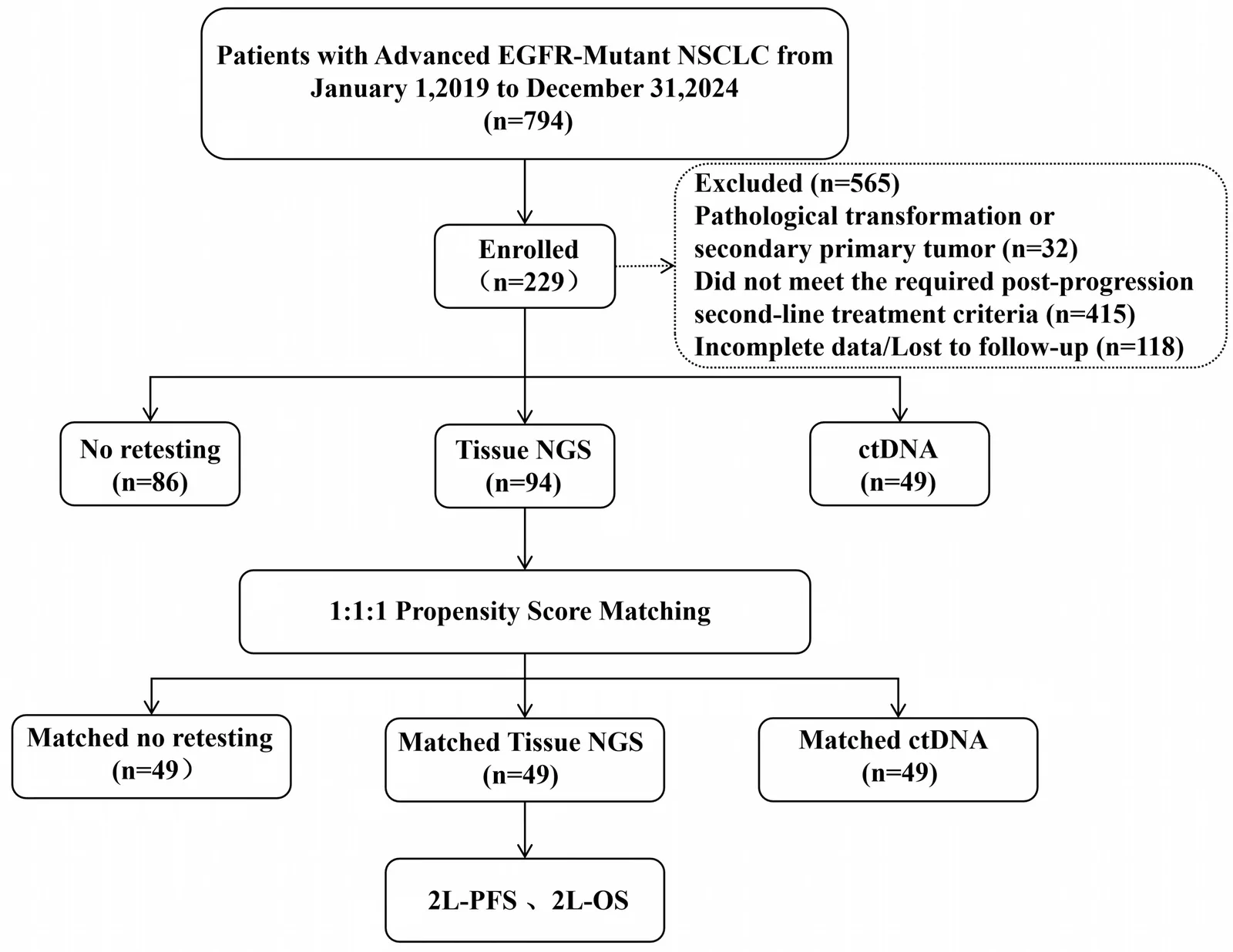
Patient selection and study flowchart. Patients with advanced EGFR-mutant NSCLC treated between January 2019 and December 2024 were screened. After applying the inclusion and exclusion criteria, 229 eligible patients were enrolled and categorized into three groups according to post-resistance testing strategies: no retesting (n = 86), tissue NGS (n = 94), and ctDNA testing (n = 49). A 1:1:1 propensity score matching (PSM) analysis was subsequently performed, resulting in 49 matched patients in each group. Survival analyses of second-line progression-free survival (2L-PFS) and overall survival from initiation of second-line therapy (2L-OS) were conducted in both the overall and matched cohorts.

The inclusion criteria were as follows: (1) age ≥18 years; (2) histologically or cytologically confirmed stage IIIB–IVB NSCLC; (3) presence of an isolated EGFR exon 19 deletion or L858R mutation at initial diagnosis; (4) radiologically confirmed disease progression after first-line EGFR-TKI treatment; and (5) availability of complete medical records.

The exclusion criteria were as follows: (1) histologic transformation or the presence of another primary malignancy; (2) absence of an evaluable post-progression second-line systemic treatment course; and (3) incomplete key clinical data or loss to follow-up.

### 2.2 Data collection, study endpoints, and patient grouping

The primary study endpoints were second-line progression-free survival (2L-PFS) and overall survival from the initiation of second-line therapy (2L-OS). Patients without documented progression or death were censored at the date of their last radiological assessment for 2L-PFS. Patients who remained alive were censored at the date of last follow-up for 2L-OS. The data cutoff date was December 2025. Tumor response and disease progression were assessed according to the Response Evaluation Criteria in Solid Tumors version 1.1 (RECIST v1.1) (14). 2L-PFS was defined as the time from initiation of second-line therapy to radiologically confirmed disease progression or death from any cause, whichever occurred first. 2L-OS was defined as the time from initiation of second-line therapy to death from any cause.

Secondary endpoints were time to treatment initiation (TTI) and subsequent treatment patterns. TTI was defined. as the interval between radiologically confirmed progression on first-line EGFR-TKIs and the initiation of second-line systemic therapy.

Clinical data collected included age, sex, smoking history, Eastern Cooperative Oncology Group performance status (ECOG PS), history of brain metastases, baseline EGFR mutation subtype, molecular profiling results obtained after EGFR-TKI resistance, first-line progression-free survival (PFS), and systemic treatment regimens.

According to the post-resistance molecular testing strategy relevant to the initiation of second-line therapy, patients were categorized into three mutually exclusive groups:

1. No-retesting group: Patients who did not undergo molecular retesting after progression on first-line EGFR-TKIs. In the absence of post-resistance molecular testing, subsequent treatment decisions were made according to routine clinical practice, incorporating available clinical characteristics, prior treatment history, and physician judgment.
2. Tissue NGS group: Patients who underwent tissue re-biopsy followed by NGS after progression on first-line EGFR-TKI therapy.
3. ctDNA group: Patients who underwent plasma ctDNA testing after progression on first-line EGFR-TKI therapy. Molecular testing results were available to the treating physicians and could be used to inform subsequent treatment selection. For patients who underwent more than one molecular test during their disease course, group classification was based on the post-resistance testing strategy relevant to the initiation of second-line therapy. No patient underwent both tissue NGS and plasma ctDNA testing before the initiation of second-line therapy. Molecular testing performed after the initiation of second-line therapy did not alter the original group assignment.

For the additional analysis linking molecular findings to subsequent treatment, post-resistance molecular results were matched to the second-line systemic regimen. Molecularly matched targeted therapy was defined as treatment directed against an actionable alteration identified by post-resistance molecular testing. Treatment categories were mutually exclusive, with patients harboring both T790M and another actionable alteration classified according to the alteration informing treatment selection.

### 2.3 Molecular testing

For patients who underwent molecular retesting after EGFR-TKI resistance, either tumor tissue or peripheral blood samples were collected according to clinical feasibility and transferred to a designated central laboratory for NGS detection. In the tissue NGS group, genomic DNA was extracted from formalin-fixed paraffin-embedded (FFPE) tumor tissue samples obtained by re-biopsy. Tissue sequencing was performed using a targeted 448-gene panel on the Illumina NextSeq 500 platform, with an average sequencing depth of approximately 2000×. For tissue samples, a minimum tumor cell content of 20% was required for sequencing. In the ctDNA group, cell-free DNA (cfDNA) was extracted from plasma samples. Plasma cfDNA sequencing was performed using the same targeted 448-gene panel and sequencing platform, with an average sequencing depth of 5000×. DNA libraries were prepared through fragmentation, end-repairing, adapter ligation, library amplification, and hybridization capture. The panel covered drug-related hotspot regions, including 7007 critical exons in 448 genes, 59 introns in 26 fusion-related genes, and promoter regions. Sequencing data were processed using the validated bioinformatics pipeline provided with the assay. All tissue and plasma samples included in the molecular testing groups passed prespecified quality-control criteria and yielded interpretable sequencing results. A molecularly negative result was defined as a technically successful assay in which no resistance-related alteration was identified. Post-resistance genomic alterations of interest included EGFR T790M and C797S, MET amplification, ERBB2 alterations, RET/ALK/ROS1 fusions, and alterations involving the PI3K–MAPK pathway. Any resistance-related alteration was defined as the presence of at least one of these alterations. TP53 was analyzed as a co-occurring alteration and was not counted toward the composite endpoint of any resistance-related alteration.

### 2.4 Statistical analysis

Statistical analyses were performed using SPSS version 27.0 (IBM Corp., Armonk, NY, USA) and GraphPad Prism version 10.1.2 (GraphPad Software, San Diego, CA, USA). Categorical variables were compared using Pearson’s chi-square test when no expected cell count was <1 and no more than 20% of expected cell counts were <5; otherwise, Fisher’s exact test was used, with the Fisher–Freeman–Halton exact test applied to contingency tables larger than 2 × 2. Continuous variables were assessed for distributional characteristics. Normally distributed variables are presented as mean ± standard deviation and were compared using one-way analysis of variance, whereas non-normally distributed variables are presented as median and interquartile range and were compared using the Kruskal–Wallis test. Survival outcomes were estimated using the Kaplan–Meier method and compared using two-sided log-rank tests. Median follow-up duration was estimated using the reverse Kaplan–Meier method.

To reduce potential confounding among the three groups, a 1:1:1 propensity score matching (PSM) analysis was performed (15). Propensity scores were estimated using a multinomial logistic regression model including age, sex, smoking history, ECOG performance status, histological subtype, brain metastases, baseline EGFR mutation subtype, generation of first-line EGFR-TKIs, and first-line PFS. Nearest-neighbor matching without replacement was applied using a caliper width of 0.2 standard deviations of the logit of the propensity score. Covariate balance was assessed using standardized mean differences (SMDs).

Exploratory subgroup analyses were performed using univariable, unadjusted Cox proportional hazards models to compare 2L-PFS between the ctDNA and tissue NGS groups within each clinical subgroup. The proportional hazards assumption was assessed using residual-based and time-dependent diagnostics for the overall and subgroup models. No evidence of non-proportionality was observed for the overall comparison, although potential non-proportionality could not be excluded in an individual subgroup. A *post hoc* sensitivity analysis of TTI was also performed after excluding patients with TTI >90 days to assess whether extreme delay values materially influenced the between-group comparison. To assess the potential influence of variation in treatment initiation timing, sensitivity analyses were conducted among patients who initiated second-line therapy within 28 days after radiologically confirmed progression, with a 30-day window used for supplementary validation. All statistical tests were two-sided, and P <0.05 was considered statistically significant. No formal adjustment for multiple comparisons was applied; therefore, P values from secondary, pairwise, sensitivity, and subgroup analyses should be interpreted as nominal and exploratory.

## 3 Results

### 3.1 Patient selection and baseline characteristics

A total of 229 patients with advanced EGFR-mutant NSCLC who developed disease progression after first-line EGFR-TKI treatment were included in this study, including 86 patients in the no-retesting group, 94 in the tissue NGS group, and 49 in the ctDNA group. Baseline clinical characteristics are summarized in **Table 1**. Before matching, baseline EGFR mutation subtype differed significantly among the three groups (P = 0.028), whereas no significant differences were observed in age, sex, smoking history, histological subtype, brain metastases, ECOG PS, first-line EGFR-TKI generation, or first-line PFS (all P > 0.05).

**Table 1:** Baseline clinical characteristics of patients before and after 1:1:1 propensity score matching.

Variables	Before PSM				After 1:1:1 PSM			
	No retesting (n=86)	Tissue NGS (n=94)	ctDNA (n=49)	P value	No retesting (n=49)	Tissue NGS (n=49)	ctDNA (n=49)	P value
Age, Mean ± SD	57.7 ± 9.9	57.2 ± 11.6	58.3 ± 10.4	0.897	58.1 ± 9.2	58.8 ± 12.8	58.3 ± 10.4	0.916
Sex, n (%)				0.139				0.971
Female	41 (47.7%)	52 (55.3%)	32 (65.3%)		31 (63.3%)	31 (63.3%)	32 (65.3%)	
Male	45 (52.3%)	42 (44.7%)	17 (34.7%)		18 (36.7%)	18 (36.7%)	17 (34.7%)	
Smoking history, n (%)				0.880				0.861
No	69 (80.2%)	76 (80.9%)	41 (83.7%)		42 (85.7%)	40 (81.6%)	41 (83.7%)	
Yes	17 (19.8%)	18 (19.1%)	8 (16.3%)		7 (14.3%)	9 (18.4%)	8 (16.3%)	
ECOG at progression, n (%)				0.353				0.827
0-1	80 (93.0%)	86 (91.5%)	42 (85.7%)		44 (89.8%)	43 (87.8%)	42 (85.7%)	
≥2	6 (7.0%)	8 (8.5%)	7 (14.3%)		5 (10.2%)	6 (12.2%)	7 (14.3%)	
Histology, n (%)				0.810				0.871
Adenocarcinoma	82 (95.3%)	91 (96.8%)	48 (98.0%)		46 (93.9%)	47 (95.9%)	48 (98.0%)	
Non-adenocarcinoma	4 (4.7%)	3 (3.2%)	1 (2.0%)		3 (6.1%)	2 (4.1%)	1 (2.0%)	
Brain metastasis, n (%)				0.687				0.901
Absent	67 (77.9%)	72 (76.6%)	35 (71.4%)		37 (75.5%)	36 (73.5%)	35 (71.4%)	
Present	19 (22.1%)	22 (23.4%)	14 (28.6%)		12 (24.5%)	13 (26.5%)	14 (28.6%)	
Initial mutation type, n (%)				0.028*				0.158
19del	38 (44.2%)	58 (61.7%)	31 (63.3%)		22 (44.9%)	29 (59.2%)	31 (63.3%)	
L858R	48 (55.8%)	36 (38.3%)	18 (36.7%)		27 (55.1%)	20 (40.8%)	18 (36.7%)	
First-line TKI generation, n (%)				0.271				0.347
1st-generation	71 (82.6%)	84 (89.4%)	46 (93.9%)		40 (81.6%)	43 (87.8%)	46 (93.9%)	
2nd-generation	2 (2.3%)	3 (3.2%)	0 (0.0%)		2 (4.1%)	2 (4.1%)	0 (0.0%)	
3rd-generation	13 (15.1%)	7 (7.4%)	3 (6.1%)		7 (14.3%)	4 (8.2%)	3 (6.1%)	
First-line mPFS (months)	10.0 (5.2-20.8)	14.0 (8.2-21.0)	12.0 (7.0-18.0)	0.066	11.0 (6.0-19.0)	14.0 (8.0-19.0)	12.0 (7.0-18.0)	0.546

Data are presented as mean ± SD, median (IQR), or n (%). Continuous variables were compared using the Kruskal–Wallis test. Categorical variables were compared using Pearson’s chi-square test, except for histological subtype and first-line EGFR-TKI generation, which were analyzed using the Fisher–Freeman–Halton exact test.

To further reduce the potential influence of baseline differences on outcome comparisons, a 1:1:1 propensity score matching (PSM) analysis was performed. After matching, 147 patients were included in the matched cohort, with 49 patients in each group. Covariate balance improved after matching, and the SMDs before and after matching are shown in **Supplementary Figure S1**.

### 3.2 Time to treatment initiation

As shown in **Figure 2A**, median TTI differed significantly among the three groups in the overall cohort (P < 0.001). Median TTI was 10.5 days (IQR, 6.0–22.8) in the no-retesting group, 20.5 days (IQR, 12.3–32.8) in the tissue NGS group, and 11.0 days (IQR, 8.0–21.0) in the ctDNA group. Pairwise comparisons demonstrated significantly longer TTI in the tissue NGS group than in both the no-retesting and ctDNA groups (both P < 0.001), whereas no significant difference was observed between the no-retesting and ctDNA groups (P = 0.372).

**Figure 2:**
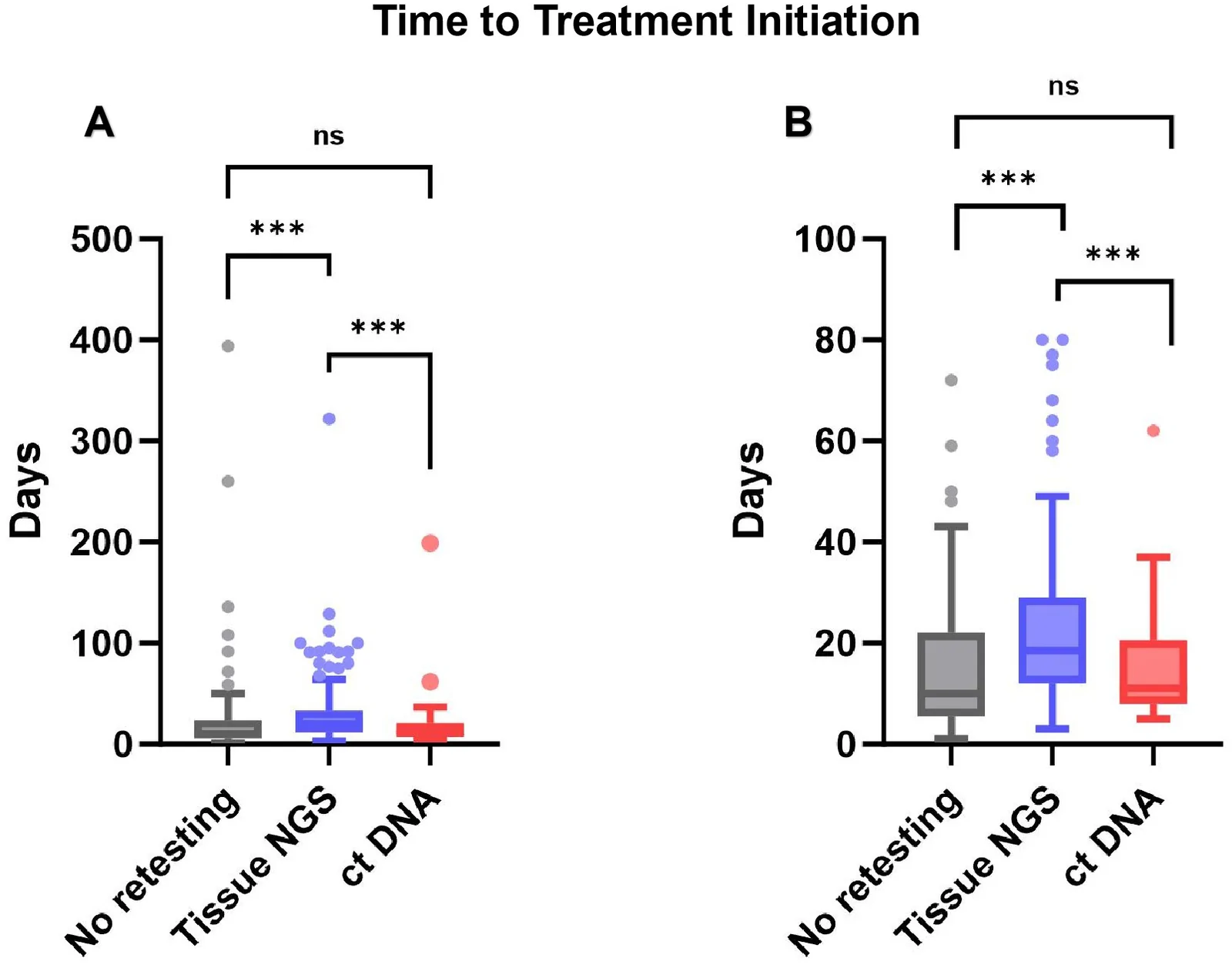
Comparison of time to treatment initiation (TTI) among different post-resistance molecular testing strategies. **(A)** TTI in the overall cohort. **(B)** *Post hoc* sensitivity analysis after excluding patients with TTI >90 days. Boxes represent the interquartile range (IQR), center lines indicate the median, and whiskers indicate the minimum and maximum values. ***P < 0.001; ns, not significant.

Because several patients had unusually prolonged TTI values, a *post hoc* sensitivity analysis was performed after excluding 16 patients with TTI >90 days (5 in the no-retesting group, 10 in the tissue NGS group, and 1 in the ctDNA group) to assess the influence of these extreme values on the between-group comparison. As shown in **Figure 2B**, the differences in median TTI remained significant among the three groups (P < 0.001). Median TTI was 10.0 days (IQR, 6.0–22.0), 18.5 days (IQR, 12.0–29.0), and 11.0 days (IQR, 8.0–19.5) in the no-retesting, tissue NGS, and ctDNA groups, respectively. Consistent with the primary analysis, TTI remained significantly longer in the tissue NGS group than in both the no-retesting and ctDNA groups (both P < 0.001), whereas no significant difference was observed between the no-retesting and ctDNA groups (P = 0.181).

### 3.3 Post-resistance molecular profiles detected by tissue NGS and ctDNA testing

To compare resistance mechanisms identified by different testing modalities, molecular alterations detected in the tissue NGS group (n = 94) and the ctDNA group (n = 49) were analyzed (**Figure 3**; **Table 2**). All 94 tissue NGS and 49 ctDNA assays included in the analysis yielded technically successful and interpretable sequencing results. Resistance-related alterations were identified in 66.0% (62/94) and 77.6% (38/49) of patients, respectively; the remaining 32 (34.0%) and 11 (22.4%) patients had molecularly negative results. The difference in the detection of resistance-related alterations between groups was not statistically significant (P = 0.181). EGFR T790M was identified in 52.1% (49/94) of patients in the tissue NGS group and 67.3% (33/49) of patients in the ctDNA group (P = 0.109). For selected structural or copy-number–related resistance mechanisms, tissue NGS showed numerically higher detection rates. MET amplification was detected in 7.4% (7/94) of patients in the tissue NGS group and 2.0% (1/49) in the ctDNA group (P = 0.264). RET/ALK/ROS1 fusions were detected in 3.2% (3/94) and 2.0% (1/49) of patients, respectively (P = 1.000). No significant differences were observed in the frequencies of TP53 co-mutations, EGFR C797S, HER2 alterations, or downstream pathway alterations between the two groups (all P > 0.05). No statistically significant differences in the frequencies of the evaluated post-resistance genomic alterations were observed between tissue NGS and ctDNA testing.

**Figure 3:**
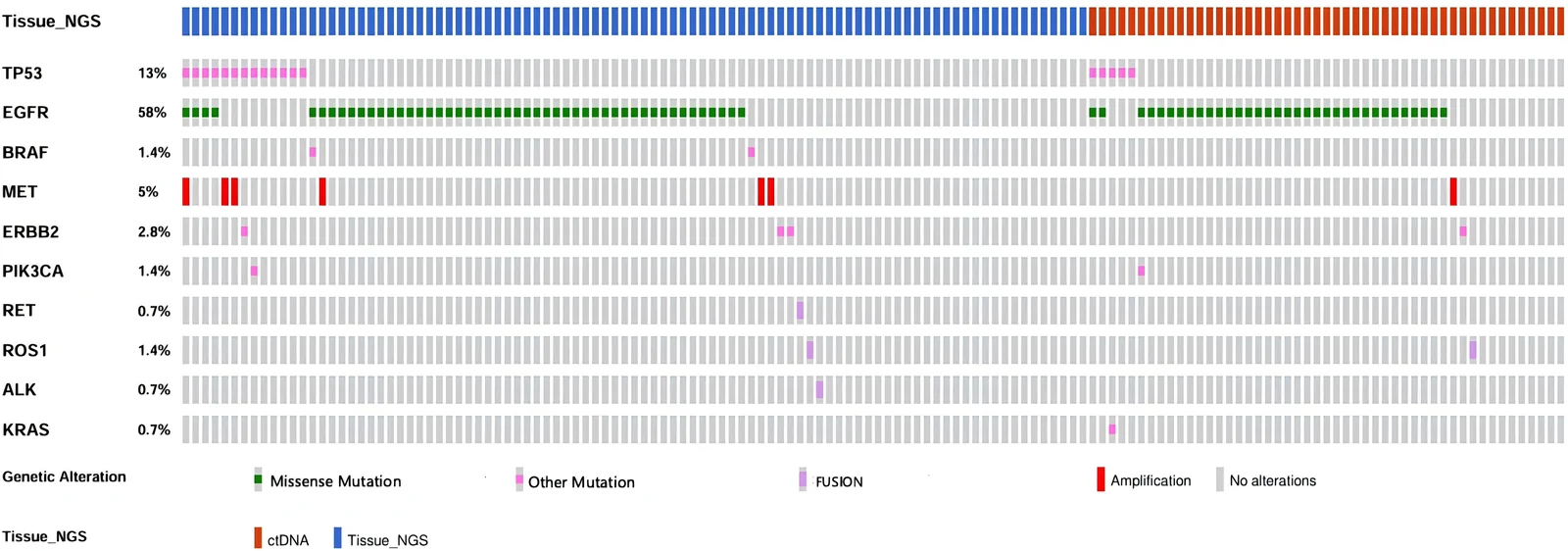
Oncoprint of acquired resistance alterations identified by tissue NGS and ctDNA testing. Each column represents an individual patient, and each row represents a specific genomic alteration. The top annotation bar indicates the testing modality (tissue NGS or ctDNA). Different colors denote distinct types of genomic alterations, including missense mutations, amplifications, fusions, and other alterations. Gray boxes indicate the absence of detectable alterations.

**Table 2:** Post-resistance genomic alterations detected by tissue NGS and ctDNA testing.

Alteration	Tissue NGS (n=94)	ctDNA (n=49)	P value
Any resistance-related alteration	62 (66.0%)	38 (77.6%)	0.181
T790M	49 (52.1%)	33 (67.3%)	0.109
TP53	14 (14.9%)	5 (10.2%)	0.605
C797S	3 (3.2%)	2 (4.1%)	1.000
MET	7 (7.4%)	1 (2.0%)	0.264
ERBB2	3 (3.2%)	1 (2.0%)	1.000
Oncogenic fusions			
RET/ALK/ROS1	3 (3.2%)	1 (2.0%)	1.000
Bypass/downstream pathways			
PIK3CA/BRAF/KRAS	4 (4.3%)	2 (4.1%)	1.000

Data are presented as n (%). P values were calculated using Pearson’s chi-square test or Fisher’s exact test, as appropriate. Any resistance-related alteration included EGFR T790M/C797S, MET amplification, ERBB2 alterations, RET/ALK/ROS1 fusions, and PI3K–MAPK pathway alterations; TP53 alone was not counted toward this composite endpoint. Patients with co-occurring alterations were counted in each relevant category; therefore, percentages do not sum to 100%.

### 3.4 Distribution of second-line treatment strategies

Second-line treatment patterns were compared among the three post-resistance testing groups (**Figure 4**). When classified according to whether targeted therapy was included, treatment patterns differed significantly among the groups (P = 0.039). The proportions of patients receiving targeted therapy were 81.4% (70/86), 89.4% (84/94), and 95.9% (47/49) in the no-retesting, tissue NGS, and ctDNA groups, respectively. Corresponding rates of non-targeted therapy were 18.6% (16/86), 10.6% (10/94), and 4.1% (2/49). Within the no-retesting group, 51 of 86 patients (59.3%) received an empiric EGFR-TKI-containing regimen without post-resistance molecular confirmation, including osimertinib-containing therapy in 35 patients (40.7%), other third-generation EGFR-TKIs in 3 patients (3.5%), and first- or second-generation EGFR-TKIs in 13 patients (15.1%). An additional 4 patients (4.7%) received antiangiogenic monotherapy, 15 patients (17.4%) received antiangiogenic therapy combined with chemotherapy or immunotherapy, and 16 patients (18.6%) received non-targeted treatment. Pairwise comparison showed a higher rate of targeted therapy use in the ctDNA group than in the no-retesting group (95.9% vs. 81.4%, P = 0.018).

**Figure 4:**
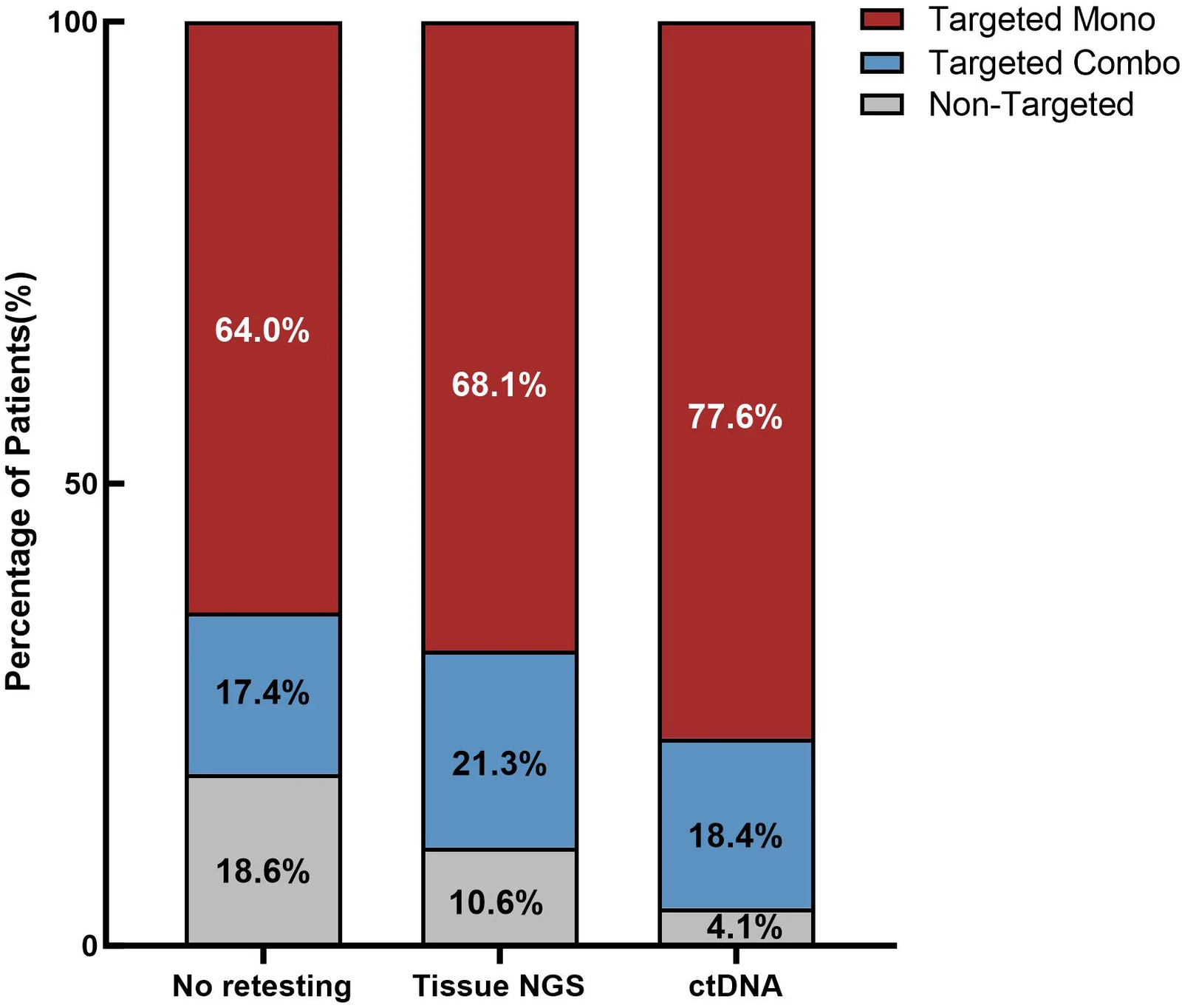
Distribution of second-line treatment patterns across different post-resistance molecular testing strategies. Second-line treatments were categorized as targeted monotherapy, targeted combination therapy, or non-targeted therapy. The proportions of each treatment category are shown for the no-retesting, tissue NGS, and ctDNA groups.

When further categorized as targeted monotherapy, targeted combination therapy, or non-targeted therapy, the overall distribution did not differ significantly among groups (P = 0.140). However, the ctDNA group showed the highest proportion of targeted monotherapy (77.6%) and the lowest proportion of non-targeted therapy (4.1%).

Further linkage of post-resistance molecular findings with second-line treatment showed that among T790M-positive patients, 45/49 (91.8%) in the tissue NGS group and 30/33 (90.9%) in the ctDNA group received a third-generation EGFR-TKI-containing regimen. Of these, osimertinib-containing therapy was administered to 43/49 (87.8%) and 29/33 (87.9%), respectively. Patients with selected non-T790M actionable alterations received molecularly matched targeted therapy, whereas targeted therapy-containing regimens were also used in some patients without an identified actionable alteration (**Table 3**).

**Table 3:** Relationship between post-resistance molecular findings and subsequent second-line treatment.

Post-resistance molecular finding	Subsequent second-line treatment	Tissue NGS (n=94), n (%)	ctDNA (n=49), n (%)
T790M detected	Osimertinib monotherapy	33 (35.1)	24 (49.0)
	Osimertinib-based combination therapy	10 (10.6)	5 (10.2)
	Other third-generation EGFR-TKI (aumolertinib/furmonertinib)	2 (2.1)	1 (2.0)
	Other EGFR-TKI-based regimens	2 (2.1)	1 (2.0)
T790M with another actionable alteration detected*	Treatment matched to co-occurring actionable alteration	2 (2.1)	2 (4.1)
Other actionable alteration detected (without T790M)	Molecularly matched targeted therapy	6 (6.4)	1 (2.0)
	Other targeted therapy-containing regimens	5 (5.3)	2 (4.1)
No actionable alteration detected	Targeted therapy-containing regimens	24 (25.5)	11 (22.4)
	Non-targeted therapy	10 (10.6)	2 (4.1)

Data are presented as n (%). Treatment categories were mutually exclusive. Molecularly matched targeted therapy was defined as treatment directed against an actionable alteration identified by post-resistance molecular testing. Targeted therapy was defined as a regimen containing an EGFR-TKI or an antiangiogenic agent. Targeted monotherapy referred to the use of a targeted agent without chemotherapy or immunotherapy, whereas targeted combination therapy included a targeted agent combined with chemotherapy, immunotherapy, or another targeted agent. *Patients with co-occurring T790M and another actionable alteration were classified separately according to the alteration informing treatment selection. NGS, next-generation sequencing; ctDNA, circulating tumor DNA; EGFR-TKI, epidermal growth factor receptor tyrosine kinase inhibitor.

### 3.5 Survival outcomes

To evaluate the association between post-resistance testing strategies and clinical outcomes, Kaplan–Meier analyses were performed in the overall cohort (**Figures 5A, B**). The median follow-up duration estimated using the reverse Kaplan–Meier method was 47.5 months (range, 1.5–81.3) in the no-retesting group, 52.4 months (range, 0.6–83.5) in the tissue NGS group, and 48.0 months (range, 1.0–55.3) in the ctDNA group. Deaths occurred in 67 (77.9%), 73 (77.7%), and 42 (85.7%) patients, respectively. In the unmatched cohort (n = 229), the median 2L-PFS was 9.0 months in both the tissue NGS and ctDNA groups, compared with 6.0 months in the no-retesting group (P = 0.370 for tissue NGS vs. ctDNA). The median 2L-OS was 20.0, 21.0, and 12.0 months in the tissue NGS, ctDNA, and no-retesting groups, respectively. Pairwise comparisons showed that 2L-OS was significantly longer in the ctDNA group than in the no-retesting group (P = 0.030), whereas no significant difference was observed between the tissue NGS and no-retesting groups (P = 0.340) or between the ctDNA and tissue NGS groups (P = 0.183).

**Figure 5:**
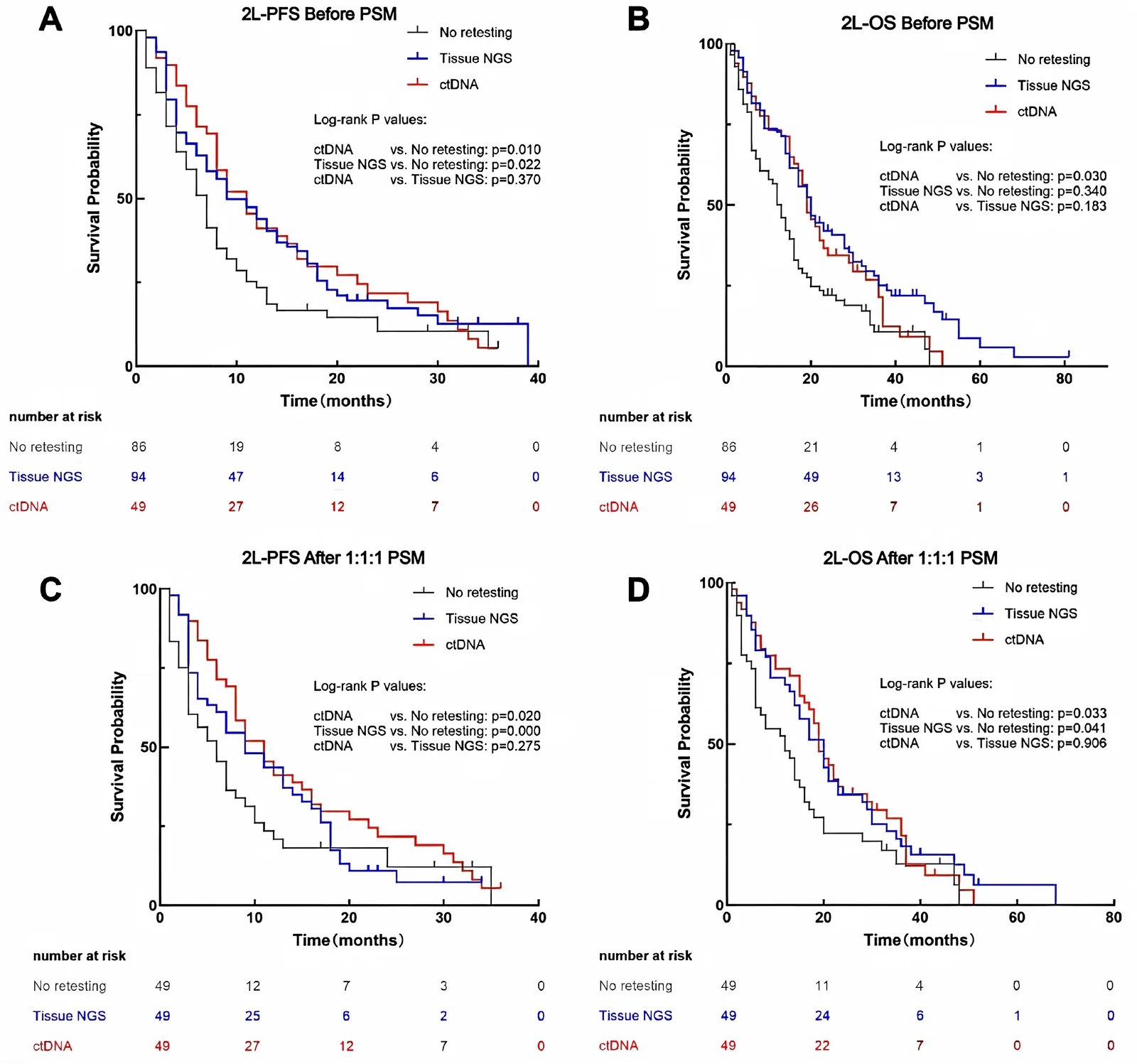
Kaplan–Meier curves of clinical outcomes according to post-resistance molecular testing strategy. **(A)** 2L-PFS in the overall cohort. **(B)** 2L-OS in the overall cohort. **(C)** 2L-PFS in the propensity score–matched cohort. **(D)** 2L-OS in the propensity score–matched cohort.

A 1:1:1 propensity score–matched analysis was subsequently performed (**Figures 5C, D**). After matching, 147 patients were included (49 per group). The median 2L-PFS was 5.0, 9.0, and 9.0 months in the no-retesting, tissue NGS, and ctDNA groups, respectively (overall P = 0.005). The corresponding median 2L-OS values were 12.0, 20.0, and 19.0 months (overall P = 0.074).

Because TTI was significantly longer in the tissue NGS group than in the ctDNA group, additional sensitivity analyses were performed by restricting the cohort to patients who initiated second-line treatment within 28 days of radiographic progression, with a 30-day window used for validation. Under both restrictions, no significant differences in 2L-PFS or 2L-OS were observed between the tissue NGS and ctDNA groups (**Supplementary Table S1**).

### 3.6 Exploratory subgroup analysis: ctDNA versus tissue NGS

To explore the association of testing strategy with 2L-PFS across different clinical subgroups, an exploratory subgroup analysis was performed using 2L-PFS as the endpoint (**Figure 6**). Cox proportional hazards models showed hazard ratios close to unity across most predefined subgroups, with 95% confidence intervals crossing 1. A nominal association was observed in patients aged ≥65 years (HR = 0.346, 95% CI: 0.148–0.809; P = 0.014). However, several subgroups contained few patients and yielded wide confidence intervals, resulting in imprecise and potentially unstable effect estimates. Potential non-proportionality in an individual subgroup further limited the interpretation of subgroup-specific estimates. Formal interaction testing was therefore not performed, and no adjustment for multiple comparisons was applied. Accordingly, all subgroup findings should be considered exploratory and hypothesis-generating rather than evidence of differential associations across subgroups.

**Figure 6:**
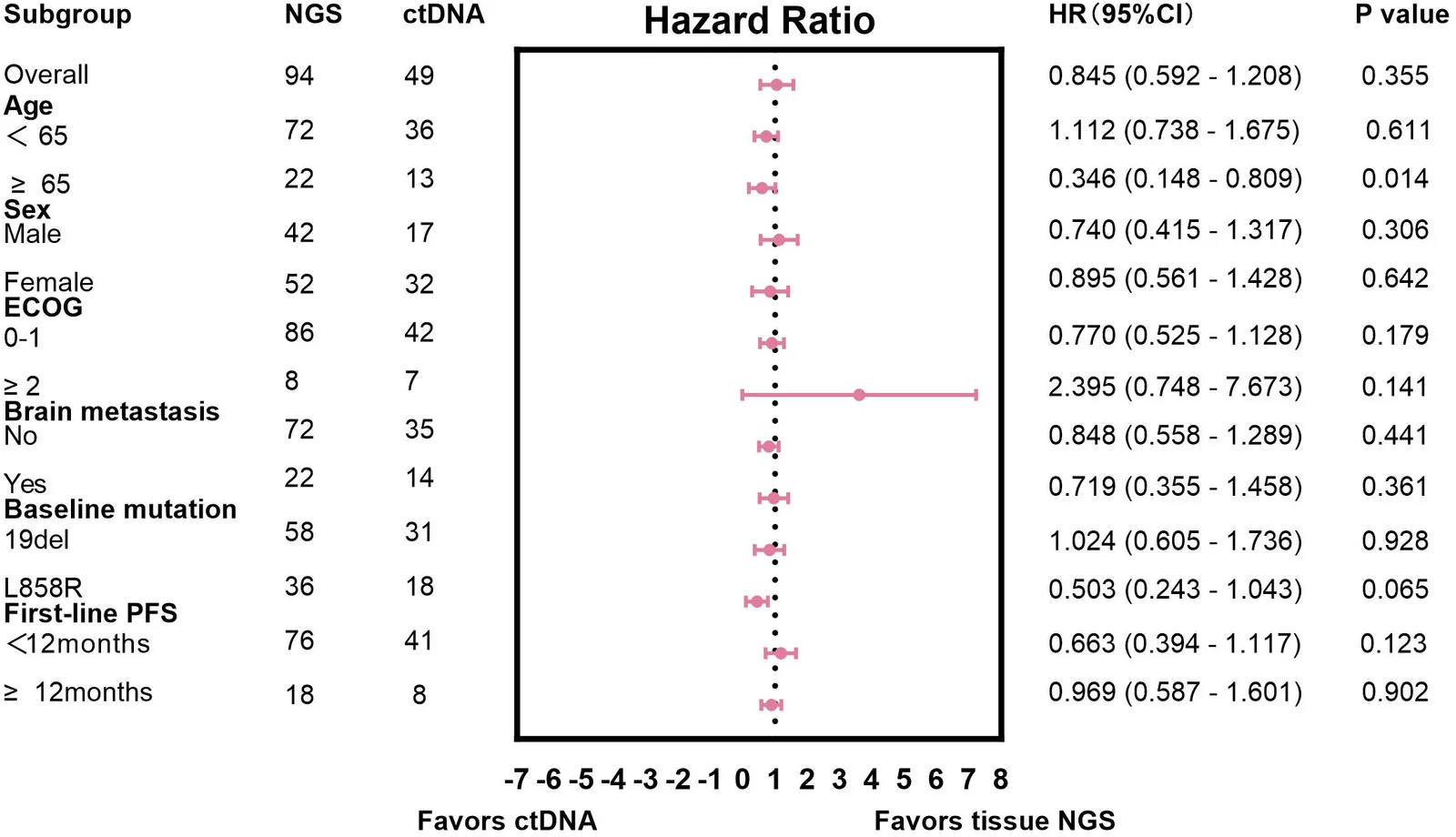
Exploratory subgroup analysis of 2L-PFS comparing ctDNA and tissue NGS. Forest plot showing hazard ratios (HRs) and 95% confidence intervals (CIs) for 2L-PFS across predefined clinical subgroups. An HR <1 indicates a lower estimated hazard in the ctDNA group relative to the tissue NGS group, whereas an HR >1 indicates a higher estimated hazard. HRs and corresponding 95% CIs were estimated using Cox proportional hazards models.

## 4 Discussion

This real-world study evaluated post-resistance molecular testing strategies in patients with EGFR-mutant advanced NSCLC after progression on first-line EGFR-TKI therapy. For the primary survival endpoints, longer 2L-PFS was observed in the molecularly retested groups than in the no-retesting group, whereas no statistically significant differences in 2L-PFS or 2L-OS were observed between the ctDNA and tissue NGS groups. For the secondary endpoints, ctDNA testing was associated with a shorter TTI than tissue NGS, and patients who underwent molecular retesting had a higher observed proportion of subsequent targeted therapy use than those without retesting. Together, these findings characterize both survival outcomes and treatment delivery across post-resistance molecular testing strategies in routine clinical practice.

In our cohort, the median TTI was nearly 10 days shorter in the ctDNA group than in the tissue NGS group. This difference may be clinically relevant in the post-resistance setting, where disease progression and deterioration in performance status can occur during treatment delays. Previous studies have linked delayed cancer treatment with worse outcomes across several tumor types (16, 17). Tissue re-biopsy often involves lesion selection, procedural scheduling, tissue acquisition, pathological review, and sequencing (7). Plasma ctDNA testing avoids the procedural steps required for tissue re-biopsy and has been associated with shorter molecular testing turnaround times in previous studies (18). This is consistent with plasma-based testing strategies that aim to shorten molecular testing turnaround time and accelerate treatment selection (9, 12). In the present study, however, assay-specific turnaround time was not separately recorded. Therefore, the observed TTI difference reflects the entire clinical pathway from radiographic progression to second-line treatment initiation and cannot be attributed solely to sequencing turnaround time. Notably, TTI did not differ significantly between the ctDNA and no-retesting groups.

The ctDNA group had the highest observed proportion of subsequent targeted therapy use. In the ctDNA group, 95.9% of patients received subsequent targeted therapy, compared with 89.4% in the tissue NGS group and 81.4% in the no-retesting group. This difference is important in routine practice. When molecular results are delayed or tissue samples are insufficient, treatment decisions may need to be made before complete molecular information becomes available (9). Earlier availability of molecular results may reduce this gap and increase the chance of genotype-matched therapy (19, 20). In our study, however, overall targeted therapy use did not necessarily represent molecularly matched treatment. The additional treatment-linkage analysis showed that more than 90% of T790M-positive patients in both molecularly retested groups received a third-generation EGFR-TKI-containing second-line regimen, while selected patients with other actionable alterations received corresponding molecularly matched treatment (**Table 3**). Similar findings have been reported in studies of plasma-based genotyping, in which blood-based testing shortened molecular testing time and improved access to biomarker-guided treatment (9, 13, 21).

The two testing strategies also differed in the resistance alterations they identified. The overall detection rate of resistance-related alterations was numerically higher in the ctDNA group than in the tissue NGS group (77.6% vs. 66.0%), although the difference did not reach statistical significance. Plasma ctDNA may capture tumor-derived DNA released from multiple disease sites and can therefore provide complementary information on tumor heterogeneity (10, 11, 22). In contrast, tissue NGS detected numerically more MET amplifications (7.4% vs. 2.0%) and fusion events (3.2% vs. 2.0%) than ctDNA, a finding that is consistent with previous reports evaluating plasma-based resistance profiling (6, 7, 23). Copy-number alterations and gene fusions can be more difficult to detect in plasma because their detection is affected by ctDNA shedding, tumor fraction, metastatic distribution, and assay design (10, 23, 24). However, because tissue NGS and ctDNA were performed in different patients rather than as paired assays, the between-group differences in alteration frequencies should not be interpreted as differences in assay sensitivity or diagnostic performance. Taken together, these findings suggest that ctDNA and tissue NGS provide complementary information for resistance profiling (6, 7). ctDNA may be useful for rapid systemic molecular assessment, whereas tissue re-biopsy remains important when MET amplification, fusion events, or other structural alterations are suspected (5, 6).

In the present study, longer 2L-PFS was observed in the molecularly retested groups than in the no-retesting group, whereas no statistically significant differences in 2L-PFS or 2L-OS were observed between the ctDNA and tissue NGS groups. Similar findings were observed in both the overall cohort and the propensity score-matched cohort, as well as in the 28-day and 30-day sensitivity analyses. The absence of a significant 2L-OS difference should be interpreted with caution. In advanced NSCLC, overall survival is influenced by multiple factors beyond the initial post-resistance treatment strategy, including subsequent lines of therapy, treatment crossover, and supportive care (25, 26). As previously reported, improvements in progression-related endpoints do not necessarily translate into an overall survival benefit, particularly when effective post-progression therapies are widely available (25, 27). Although no significant 2L-OS benefit was observed, molecularly matched treatment was administered to selected patients with actionable resistance alterations identified by post-resistance molecular testing. Given the favorable tolerability profile of targeted therapies compared with conventional cytotoxic chemotherapy, appropriately selected targeted treatment may offer clinical benefits beyond survival outcomes alone (28). Whether these advantages translate into improvements in quality of life and patient-reported outcomes requires further investigation in prospective studies (29).

In the exploratory subgroup analysis, a nominal association with a lower risk of progression was observed among patients aged ≥65 years in the ctDNA group (HR = 0.346, 95% CI: 0.148–0.809; P = 0.014). Older patients are often less suitable candidates for invasive procedures and may be more vulnerable to treatment delays (16, 30). However, given the limited subgroup sample size, absence of formal interaction testing, and multiple comparisons without multiplicity adjustment, this finding may represent a chance observation and should be considered hypothesis-generating rather than evidence of a differential benefit of ctDNA testing in older patients.

This study has several limitations. First, its single-center retrospective design was subject to confounding by indication. Although PSM balanced measured covariates, unmeasured factors influencing the decision to perform molecular retesting could not be fully addressed. The no-retesting group may have included clinically heterogeneous patients, including those for whom molecular testing was not pursued because of limited clinical feasibility, disease status, physician judgment, or other patient-related considerations. These factors may have influenced subsequent treatment selection, treatment initiation timing, and clinical outcomes, and could partly explain the inferior survival observed in this group independent of the testing strategy itself. Second, the analysis included only patients who ultimately received second-line systemic therapy. Testing data were not systematically available for excluded patients, precluding their reliable classification by post-resistance testing strategy. Therefore, the findings cannot be generalized to all patients progressing after first-line EGFR-TKI therapy. Third, dynamic ctDNA monitoring and variant allele frequency data were unavailable, and a negative ctDNA result could not exclude an underlying resistance alteration because of low tumor DNA shedding. Finally, the interpretation of 2L-OS was limited by subsequent treatments and sample size. Because most patients received a first-generation EGFR-TKI, the findings may not be directly generalizable to patients progressing after first-line third-generation EGFR-TKI therapy.

In conclusion, among patients who ultimately received second-line systemic therapy, ctDNA-based molecular retesting was associated with a shorter time to treatment initiation than tissue NGS. The ctDNA group also showed the highest observed proportion of subsequent targeted therapy use. No statistically significant differences in 2L-PFS or 2L-OS were observed between the ctDNA and tissue NGS groups. These findings indicate that the value of molecular retesting extends beyond the identification of resistance mechanisms. In routine clinical practice, timely access to molecular results may help facilitate subsequent treatment decisions and treatment delivery after disease progression. ctDNA testing may therefore be a practical option for patients who require rapid treatment selection or are unable to undergo tissue re-biopsy.

## Data Availability

The datasets presented in this article are not readily available because they contain potentially identifiable patient information and are subject to institutional and patient privacy restrictions. Requests to access the datasets should be directed to Zongyang Yu at hxkyzy2024@163.com and will be considered in accordance with applicable ethical and institutional requirements.
