# Supplementary material for: Impact of post-resistance molecular retesting strategies on treatment timeliness and clinical outcomes in EGFR-mutant advanced NSCLC: a real-world study
Source: Front Oncol. 2026 Sep 25;16:1969822. doi: 10.3389/fonc.2026.1969822 (PMC13649378; doi:10.3389/fonc.2026.1969822)
Supplement: Supplementary file 1 [file DataSheet1.docx]

**Supplementary Material**

Supplementary Figure S1. Covariate balance before and after 1:1:1 propensity score matching. Gray and blue points represent SMDs before and after matching, respectively. The red dashed line indicates the threshold for ideal balance (SMD = 0.10), whereas the orange dashed line indicates the threshold for acceptable balance (SMD = 0.20). Most covariates showed improved balance after matching.

Supplementary Table S1. Sensitivity analyses according to the interval between radiographic progression and initiation of second-line therapy.

| **Time window** | **Endpoint** | **Tissue NGS, n** | **ctDNA, n** | **Median in tissue NGS,months** | **Median in ctDNA,months** | **P value** |
| --- | --- | --- | --- | --- | --- | --- |
| ≤28 days | PFS2 | 62 | 44 | 11 | 9 | 0.637 |
| ≤28 days | OS2 | 62 | 44 | 17 | 21 | 0.379 |
| ≤30 days | PFS2 | 66 | 44 | 9 | 9 | 0.595 |
| ≤30 days | OS2 | 66 | 44 | 17 | 21 | 0.477 |

To minimize potential bias related to differences in treatment initiation timing between the ctDNA and tissue NGS groups, additional sensitivity analyses were performed among patients who initiated second-line therapy within 28 or 30 days after radiographic progression. Median survival times were estimated using the Kaplan–Meier method, and comparisons were performed using the log-rank test.
